# Effects of reprogrammed splenic CD8^+^ T-cells in vitro and in mice with spontaneous metastatic Lewis lung carcinoma

**DOI:** 10.1186/s12885-024-12203-y

**Published:** 2024-04-25

**Authors:** E. Skurikhin, N. Ermakova, M. Zhukova, E. Pan, D. Widera, L. Sandrikina, L. Kogai, O. Pershina, A. Pakhomova, V. Yu. Pan, N. Kushlinskii, A. Kubatiev, S. Morozov, A. Dygai

**Affiliations:** 1 Institute of General Pathology and Pathophysiology, 125315 Moscow, Russia; 2 Stem Cell Biology and Regenerative Medicine Group, School of Pharmacy, Whiteknights Campus, RG6 6AP Reading, UK; 3 Goldberg ED Research Institute of Pharmacology and Regenerative Medicine, Tomsk National Research Medical Centre of the Russian Academy of Sciences, Lenin, 3, 634028 Tomsk, Russia; 4 Blokhin National Medical Research Center of Oncology, 115522 Moscow, Russia; 5 Ministry of Health of the Russian Federation, Siberian State Medical University, Moskovski, 2, 634050 Tomsk, Russia

**Keywords:** Metastatic disease, Lung cancer, Lewis lung carcinoma, Reprogrammed spleen CD8^+^ T-cells, Cell therapy

## Abstract

**Background:**

Metastatic disease is a major and difficult-to-treat complication of lung cancer. Considering insufficient effectiveness of existing therapies and taking into account the current problem of lung cancer chemoresistance, it is necessary to continue the development of new treatments.

**Methods:**

Previously, we have demonstrated the antitumor effects of reprogrammed CD8^+^ T-cells (rCD8^+^ T-cells) from the spleen in mice with orthotopic lung carcinoma. Reprogramming was conducted by inhibiting the MAPK/ERK signalling pathway through MEKi and the immune checkpoint PD-1/PD-L1. Concurrently, CD8^+^ T-cells were trained in Lewis lung carcinoma (LLC) cells. We suggested that rCD8^+^ T-cells isolated from the spleen might impede the development of metastatic disease.

**Results:**

The present study has indicated that the reprogramming procedure enhances the survival and cytotoxicity of splenic CD8^+^ T-cells in LLC culture. In an LLC model of spontaneous metastasis, splenic rCD8 + T-cell therapy augmented the numbers of CD8^+^ T-cells and CD4^+^ T-cells in the lungs of mice. These changes can account for the partial reduction of tumors in the lungs and the mitigation of metastatic activity.

**Conclusions:**

Our proposed reprogramming method enhances the antitumor activity of CD8^+^ T-cells isolated from the spleen and could be valuable in formulating an approach to treating metastatic disease in patients with lung cancer.

**Supplementary Information:**

The online version contains supplementary material available at 10.1186/s12885-024-12203-y.

## Background

Metastasis is the primary cause that elevates cancer-related deaths in patients. It significantly contributes to the mortality rate among patients with lung cancer. The incidence of metastasis in lung cancer averages around 70%, and according to recent report, it can reach up to 90% [1, 2]. Approximately 50% of cases are metastatic at the time of lung cancer diagnosis. In lung cancer, metastasis foci are often found in the opposite lung [3]. Combination chemotherapy and the use of immunobiologicals have significantly improved the survival of patients with metastatic lung cancer. By 2040, patients with metastatic lung cancer are predicted to have a survival rate of 46.7% [2]. Considering insufficient effectiveness of existing therapies and taking into account the current problem of lung cancer chemoresistance, it is necessary to continue the development of new treatments.

Metastasis of a malignant tumor is a complex multi-stage process. Cancer stem cells (CSCs) play an important role in the process of metastasis. This cell population is characterized by genetic heterogeneity and it carries a large number of mutations (such as chromosomal aberrations, increased Notch gene expression, mutations in the KRAS, FGFR1, EGFR, ALK, BRAF, PIK3CA, AKT1, HER2, MEK1, NRAS, RET, ROS1 genes) which determine high metastatic potential and chemoresistance [4–6]. It is possible to increase the effectiveness of conventional therapy by targeting CSCs. The proposed approaches to manipulate CSCs include targeting the metabolic processes of cancer cells, their epigenetic regulation, and angiogenesis [7]. Another area of therapy is immunotherapy. The use of anti-CTL4 and anti-PD-1/PD-L1 antibodies in cancer vaccines has shown promising results in preclinical studies. Some of these methods have found active use in clinical practice. Thus, the use of immune checkpoint inhibitors has improved the long-term survival of patients. However, it may not be effective enough in some categories of patients [8].

CD8^+^ T-cells are key players in antitumor immunity. Normal effector activity of immune cells is an important prerequisite for the elimination of tumor cells. CSCs and components of the tumor microenvironment modulate the T-cell antitumor response through the secretion of various growth factors [9], and functional disorders of T-cells are observed in many types of cancer [10]. Therefore, it is important to search for methods aimed at restoring and enhancing T-cell antitumor immunity.

Previously, using an orthotopic Lewis lung carcinoma (LLC) model, we showed that reprogrammed CD8^+^ T-cells (rCD8^+^ T-cells) isolated from the bone marrow and spleen have antitumor potential [11, 12]. Reprogramming was performed using a MEK inhibitor and a PD-1 blocker. Based on these results, we suggested that rCD8^+^ T-cells may exhibit antimetastatic activity. In the present study, we evaluated the effects of splenic T-cells in C57Bl/6 mice in a spontaneous LLC metastasis model. Specifically, investigated the ability of splenic rCD8^+^ T-cells to maintain cytotoxic activity in immunosuppressive effect of tumors in vitro.

## Materials and methods

### Animals

Male mice C57BL/6 (age 8–10 weeks) were obtained from the nursery of the Department of Experimental Biomodels of the Tomsk National Research Medical Center (veterinary certificate available). Animals were kept in accordance with the European Convention for the Protection of Vertebrate (Strasbourg, 1986); Principles of Good Laboratory Practice (OECD, ENV/MC/CUEM (98)17, 1997). Animal procedures and study design were approved by the Ethics Committee of the Research Institute of Pharmacology and Regenerative Medicine. E.D. Goldberg of the Tomsk National Research Medical Center (protocol No. 189,092,021).

Mice were divided into 4 groups (*n* = 10 mice in each group): group 1 - intact mice; group 2 - mice with lung cancer; group 3 - lung cancer mice treated with naive CD8^+^T-cells isolated from the spleen of intact mice (nCD8^+^T-cells); group − 4 mice with lung cancer treated with reprogrammed splenic CD8^+^ T-cells isolated from intact mice (rCD8^+^ T-cells).

### Lewis lung carcinoma cell line

The Lewis lung carcinoma (LLC) cell line C57BL was used in experiments *in vivo and in vitro* (400,263 CLS Cell Lines. Service, GmbH, Köln, Germany). LLC cells were established from the spontaneous lung adenocarcinomas that occur in C57BL/6 mice.

### Model of LLC solid tumor spontaneous metastasis

Lewis lung carcinoma cells (*n* = 5 × 10^6^ cells) suspended in 100 µl of RPMI medium were injected once subcutaneously into the right axillary region of mice [13]. Within 10 days after the injection of LLC cells, tumor growth was studied every three days [14]. After 10 days of inoculation of LLC cells, the animals were euthanized by CO_2_ overdose.

### Study design

**Fig. 1:**
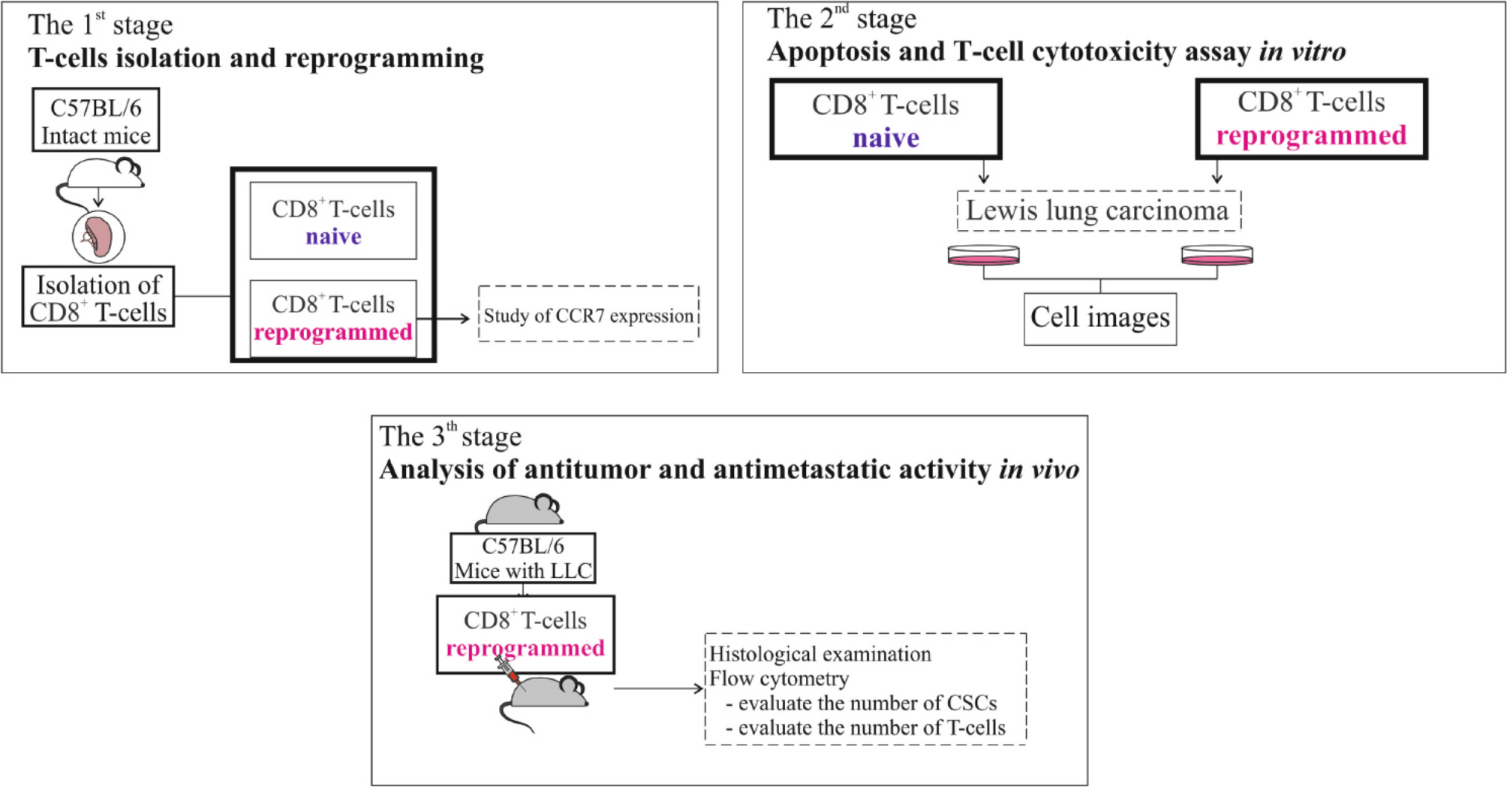
Design of study

The study design is shown in Fig. 1. At the first stage, we reprogrammed CD8^+^ T-cells obtained from the spleen of intact mice. Afterwards, the effect of the procedure was assessed - T-cell phenotype and CCP7 expression were determine. Then, to confirm the persistence of the changes generated by reprogramming, in vitro, we evaluated the effects of the exhausted procedure on C-C chemokine receptor type 7 (CCR7) expression by rCD8^+^T-cells. Apoptosis and cytotoxicity of rCD8^+^ T-cells were studied in LLC culture. Next, the morphological and histological picture of the lungs in animals treated with rCD8^+^ T-cell therapy was evaluated using the LLC metastatic model. At the final stage of the study, the content of cancer cells and CSCs, as well as CD8^+^T-cells and CD4^+^T-cells in the lungs, were studied in mice with lung cancer treated with rCD8^+^T-cell therapy. The effects of rCD8^+^ T-cells were compared with those of nCD8^+^T-cells in vitro and in vivo.

For in vivo experiments, animals were divided into intact control mice (intact control, *n* = 10), mice with LLC (pathological control, *n* = 10), mice with LLC receiving cell therapy with naive CD8^+^ T-cells (experimental group 1, *n* = 10) and rCD8^+^T-cells (experimental group 2, *n* = 10).

### Isolation of mononuclear cells

Mononuclear cells from lungs, and spleen were isolated as described earlier [15, 16]. The cell suspension was enriched with naive CD8^+^ T-lymphocytes (here and hereinafter in the text phenotype of naive CD8^+^ T-cells was defined as CD3^+^CD8^+^CD44^−^CD62L^+^) by magnetic separation. Enrichment was performed following a standard protocol using a mouse kit (EasySepTM Mouse Naive CD8 + T Cell Isolation Kit, as recommended by the manufacturer (StemCell Technologies, Vancouver, BC, Canada).

### Reprogramming of spleen CD8^+^ T-cells

Reprogramming of CD8^+^ T-cells isolated from the spleen of C57BL/6 mice was conducted using a protocol previously published [11]. In the details, cell suspension enriched with naive CD8^+^ T-cells (here and hereinafter in the text phenotype of naive CD8^+^ T-cells was defined as CD3^+^CD8^+^CD44^−^CD62L^+^) were incubated in a culture medium consisting of RPMI 1640 (Sigma-Aldrich, USA) with the addition of 10% fetal bovine serum (Sigma-Aldrich, USA), 2 mM L-glutamine (Sigma-Aldrich, USA), 10 mM HEPES (Sigma-Aldrich, USA), and 55 µM β-mercaptoethanol (Thermo Scientific™ 35602BID, USA), 37 °C, 5% CO2 for 2–3 h.

An antigen-presenting mix was prepared from LLC cell lysate by using a freeze–thaw cycle in 0.85% NaCl solution. The cycle was repeated five times in rapid succession from − 70 °C to 37 °C, and then refrozen and stored at − 70 °C before use. After the thawing, the lysate was stained by trypan blue (Sigma-Aldrich, USA). The preparation of adjuvant (Freund’s adjuvant) for the antigen-presenting mix was carried out according to the manufacturer’s standard protocol (Sigma-Aldrich, USA). Freund’s adjuvant solution was mixed with the cancer cell lysate (3 × 10^4^/mL) at a 1:1 ratio to form a thick emulsion.

For reprogramming, 50 µL of an antigen-presenting mix with 1 µM MEK inhibitor was added to a flask with CD8^+^ T-cells. The concentration of CD8^+^ T-cells was 1 × 10^8^/mL, the volume of the medium in the flask was at least 5 mL. The resulting cell suspension was incubated for 48 h at 37 °C and 5% CO2. Reprogrammed CD8^+^ T-cells were incubated for 2 h with human monoclonal antibody nivolumab at a concentration 10 µg/mL in order to protect cells from the humoral action of LLC. At the end of the incubation cycle, suspensions were washed 2 times in the medium recommended for CD8^+^ T-cells. Immunophenotype and cytotoxicity of reprogrammed CD8^+^ T-cells were analyzed with Cytation 5 (here and hereinafter in the text phenotype of reprogrammed T-cells was defined as CD8^+^CD45RA^+^CD197^hi^CD62L^+^CD95^+^).

### Exhaustion of reprogrammed splenic CD8^+^ T-cells in vitro

**Fig. 2:**
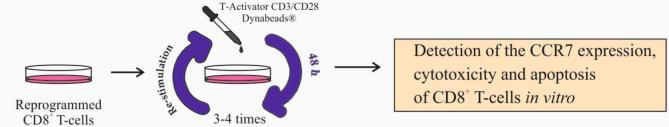
Exhaustion of reprogrammed splenic CD8^+^ T-cells in vitro

For exhaustion reprogrammed splenic CD8^+^ T-cells (rCD8^+^ T-cells) at 1–3 × 10^6^ cells/ml were in the culture medium recommended for T-lymphocytes (PBS, 2% inactivated FTS, 1 mM EDTA without Ca^2+^, Mg^2+^ and biotin) with the addition of 30 IU/ml IL-2 (Stemcell Technologies, Vancouver, USA) stimulated T-Activator CD3/CD28 Dynabeads® (Thermo Fisher Scientific Baltics, Lithuania) [17]. The ratio of particles and cells was 1:1. rCD8^+^ T-cells were incubated at 37 °C and 5% CO_2_ in the presence of MEK (10 µg/mL) or in the absence of an inhibitor. Subsequently, restimulation was performed every 48 h, a total of 3–4 times (Fig. 2). After incubation, Dynabeads were removed and CCR7 expression, apoptosis, and cytotoxicity of splenic rCD8^+^ T-cells were studied in LLC culture.

Additionally, naive splenic CD8^+^ T-cells were depleted and their activity in LLC culture was compared with rCD8^+^ T-cells.

### Detection of the CCR7 expression, cytotoxicity and apoptosis of spleen rcd8^+^ t-cells in vitro

CCR7 expression, cytotoxicity, and apoptosis of rCD8^+^ T-cells were determined according to a previously published protocol [11]. Images of cells were obtained using the cell-imaging Cytation 5 (BioTek Instruments, Inc., Winooski, VT, USA) equipped with the following cubes: DAPI (blue), GFP (green), RFP (yellow), 4× magnification.

Image analysis was performed using Gen5™ data analysis software (BioTek, Instruments, Friedrichshall, Germany) as described earlier [11].

### Introduction of naive and reprogrammed splenic CD8^+^ T-cells

To assess antimetastatic activity, naive and reprogrammed CD8^+^ T-lymphocytes were intravenously administered to recipient mice with LLC at a dose of 1 × 10^6^ cells/mouse in 0.1 ml of PBS on the 7th and 9th day of the experiment.

### Examination of laboratory animals

During the experiment, body weight, the general condition of the animal was assessed, while the fur, skin, and musculoskeletal system were examined.

### Histological examination of the lungs

Lung tissue for histological examination was fixed in 10% neutral buffered formalin and passed through solutions of alcohol and xylene of increasing concentration. Then paraffin blocks were obtained. The sections with a thickness of 3–5 microns were stained with hematoxylin and eosin [11, 18].

### Morphometric examination of the lungs

The effects of cell therapy on LLC growth were assessed by statistical comparison of the tumor node volume in the control and experimental groups at the different observation periods, according to the duration of tumor growth retardation and tumor growth inhibition index (TGII) [19]:

TGII = (Vc − Ve)/Ve × 100%,

where Vc and Ve are the average node volume in the control and experimental groups.

Linear dimensions of tumor nodes were measured in orthogonal planes and their volume was calculated in the elliptical approximation [14]. The tumor was measured with a caliper and the volume of the tumors was calculated by the formula:

V = π/6 × length × width × height.

Additionally, a weighting coefficient was determined, calculated as the ratio of the mass of the tumor in milligrams to the weight of the animal in grams.

The severity of the metastatic process was evaluated by the frequency of tumor metastasis (the percentage of animals with metastases in relation to the total number of animals in the group); degree of damage to the lungs by metastases; the average number of metastases per animal in each group; the average weight of the lungs affected by metastases; metastasis inhibition index (MII). The degree of damage to the lungs by metastases was expressed in points (Table 1).

**Table 1:** The degree of damage to the lungs by metastases

Points	Characteristics
0 points	no metastases
1 point	the number of metastases is less than 10, the diameter of metastases does not exceed 1 mm
2 points	the number of metastases from 10 to 30
3 points	the number of metastases is more than 30, metastases of various sizes
4 points	the number of metastases is less than 100, metastases without confluent growth
5 points	the number of metastases is more than 100, the presence of continuous tumor nodes

### MII was calculated using the formula

MII = ((Аc × Вc)– (Аe × Вe)) / (Аc × Вc) × 100%,

where Аc and Аe– the frequency of metastasis to the lungs in mice of the control and experimental groups, respectively, Bc and Be - the average number of metastases in the lungs per animal in the control and experimental groups, respectively [20, 21].

### Flow Cytometry

Anti-mouse monoclonal antibodies with the following specificities were used for flow cytometry: CD3 PerCP (Cat#553,067), CD4 FITC (Cat#53,046), CD8 BV510 (Cat#563,068), CD90 APC (Cat#561,409), CD62L APC (Cat#60109AD), CD95 BV421 (Cat#562,629), CD117 (c-Kit) FITC (Cat#553,354), EGF Alexa Fluor® 647 (Cat#564,226), Axl BV421 (Cat#748,028), PD-L1 (CD274) PE (Cat#558,091), CD44 APC-Cy™7, PD-1 (CD279) BV421 (Cat#748,268), CD197 (CCR7) PE (Cat#560,682) and for the intracellular staining Sox2 PE (Cat#562,195) and Ki67 APC (Cat#558,615) (all—1/50 dilution, BD Biosciences, San Jose, CA, USA). The respective isotype controls were used. All anti-mouse monoclonal antibodies were titrated to establish optimal staining dilutions. Representative flow cytometry images presented in Supplementary Fig. 1.

Staining of lung and blood mononuclears and flow cytometry was carried out as described earlier [11]. CSCs and T-cell panels are summarized in subpopulations were defined according to the markers specified in Table 2.

**Table 2:** Studied populations of CSCs and T-cells

CSCs populations	T-cells populations/Characteristic
Axl^+^	CD8^+^CD45RA^+^CD197^hi^CD62L^+^CD95^+^ / Stem cell–like memory (TSCM) CD8^+^ T cells. This population have high self-renewability, multipotency and proliferation capacity
Axl^+^Sox2^+^	CD3^+^CD4^+^CD8^−^Ki67^+^ / Proliferating CD4^+^T-cells
Axl^+^CD117^+^	CD3^+^CD4^+^Ki67^+^PD-1^+^ / Activated proliferating CD4^+^T-cells
CD44^hi^CD90^+^Sox2^+^	CD3^+^CD8^+^Ki67^+^PD-1^+^ / Activated proliferating CD8^+^T-cells
CD117^+^CD44^+^	CD3^+^CD8^+^Ki67^+^ / Proliferating CD8^+^T-cells
CD117^+^EGF^+^CD44^+^PD-L1^+^PD-1^+^	CD3^+^CD8^+^PD-1^+^ / Exhausted CD8^+^ T-cells. This population is characterized by decreased antigen-dependent cytokine secretion and increased expression of inhibitory surface receptors
CD117^+^Sox2^+^	CD4^−^CD3^−^CD8^+^CD62L^+^ / Memory CD8^+^T-cells
CD117^+^EGF^+^	
CD117^+^EGF^+^CD44^+^	
EGF^+^CD44^+^Sox2^+^	
CD117^+^EGF^+^CD44^+^Sox2^+^	

### Statistical analysis

Statistical analysis was performed by methods of variational statistics using the SPSS 12.0 software (SPSS Inc., Chicago, IL, USA). The arithmetic mean (M), error of the mean (m), and the probability value (p) were calculated. The difference between the two compared values was considered significant at *p* < 0.05.

## Results

### *Exhaustion does not affect CCR7 overexpression by rCD8*^*+*^*T-cells of the spleen*

**Fig. 3:**
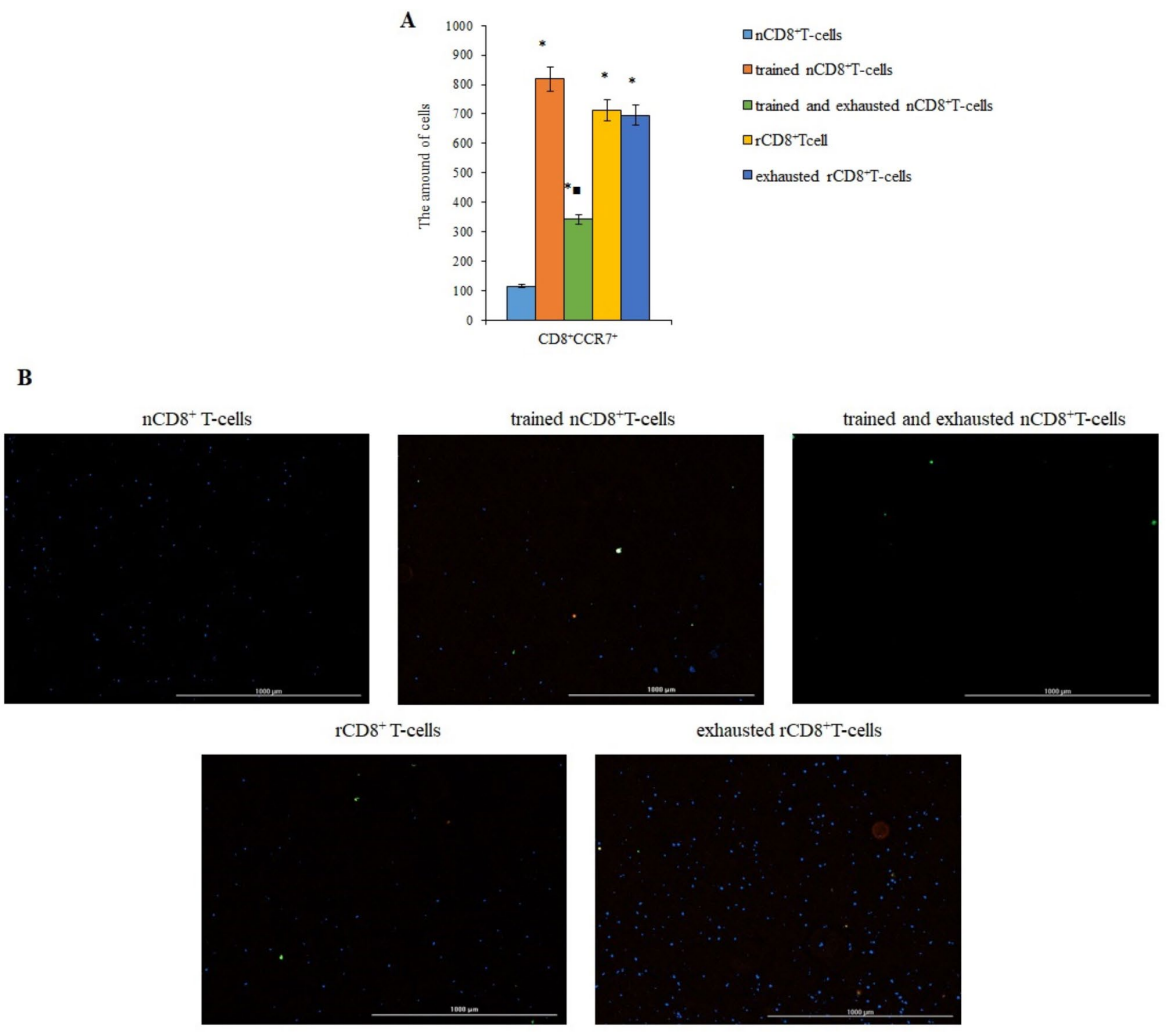
The number of CCR7^+^ T-cells in a culture of naive (nCD8^+^T-cells), trained naive (trained nCD8^+^T-cells), trained and exhausted naive (trained and exhausted nCD8^+^T-cells), reprogrammed (rCD8^+^T-cells), and exhausted reprogrammed (exhausted rCD8^+^T-cells) CD8^+^ T-cells isolated from the spleen of C57BL/6 mice. **A** The count of nCD8^+^T-cells, trained nCD8^+^T-cells, trained and exhausted nCD8^+^T-cells, rCD8^+^T-cells, and exhausted rCD8^+^T-cells of the spleen of C57BL/6 mice expressing the CCR7 marker in T-cells culture. Data were obtained by analyzing images obtained using Cytation 5 Cell Imaging Multimode Reader, widefield fluorescence microscopy.**B** 4× images of T-cells stained with: Hoechst 34,580 (blue) to identify cell nuclei; CD8 FITC (green); CCR7 AF555 (red); (Hoechst^+^CD8^+^ CCR7^+^) composite image using all three colors. Determination of the percentage of cells CD8^+^CCR7^+^ is made by the ratio of cells counted in green and red channel to total cells counted using blue (DAPI) channel. Images were obtained using Cytation 5 Cell Imaging Multimode Reader, widefield fluorescence microscopy. All scale bars are 1000 μm. *– for comparison with the naive T-cells, ■– for comparison with trained naive T-cells (Mann–Whitney test, *p*-value < 0.05)

The expression of CCR7 by these populations was significantly increased by the training of naive and reprogrammed CD8^+^ T-cells (Fig. 3). After exhaustion, the number of trained CD8^+^ T-cells expressing CCR7 decreased by 58% (here and hereinafter in the text phenotype of trained naive CD8^+^ T-cells was defined as CD3^+^CD8^+^CD44^−^CD62L^+^CD197^low^). Exhaustion had no effect on rCD8^+^ T-cells expressing CCR7 (Fig. 3).

### In vitro, splenic rCD8^+^ T-cells were more resistant to LLC action than naive and trained splenic CD8^+^ T-cells

The study of apoptosis and cytotoxicity of naive, trained naïve CD8^+^T-cells and reprogrammed CD8^+^T-cells was performed in LLC culture. The ratio of populations of CD8^+^ T-cells and LLC cells in culture was 0:1, 1:1, 2.5:1, 5:1, and 10:1, respectively.

**Fig. 4:**
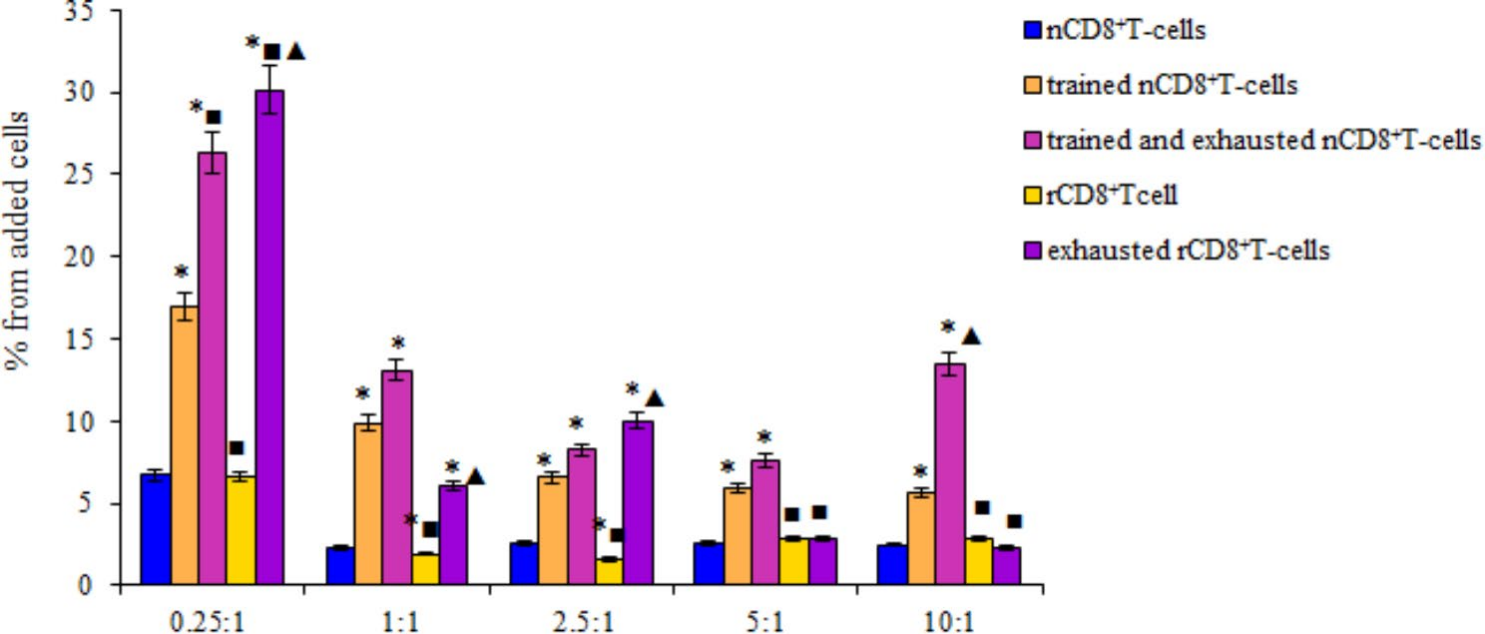
The number of apoptotic naive (nCD8^+^T-cells), trained naive (trained nCD8^+^T-cells), trained and exhausted naive (trained and exhausted nCD8^+^T-cells), reprogrammed (rCD8^+^T-cells), and exhausted reprogrammed (exhausted rCD8^+^T-cells) CD8^+^ T-cells isolated from the spleen of C57BL/6 mice after co-culturing with LLC (% from added cells) in ratio between T-lymphocytes and LLC 0.25:1, 1:1, 2.5:1, 5:1, and 10:1. *– for comparison with the naive T-cells, ■– for comparison with trained naive T-cells, ▲– for comparison with reprogrammed T-cells (Mann–Whitney test, *p*-value < 0.05). Data were obtained by analyzing images obtained using Cytation 5 Cell Imaging Multimode Reader, widefield fluorescence microscopy

Trained CD8^+^ T-cells at all concentrations are less resistant to the apoptotic action of LLC in comparison with nCD8^+^ T-cells (Fig. 4). Meanwhile, rCD8^+^T-cells were more resistant to apoptosis. The smallest number of apoptotic rCD8^+^T-cells was observed at ratios of 1:1 and 2.5:1.

Additionally, the effect of the exhausted procedure on the apoptosis of trained and rCD8^+^ T-cells in LLC culture were studied. Exhaustion increased the apoptosis of both trained naive and rCD8^+^T-cells. However, while exhaustion had a negative effect on trained CD8^+^ T-cells at all concentrations, it affected rCD8^+^T-cells at ratios of 0.25:1, 1:1, and 2.5:1 (Fig. 4).

### In LLC culture, splenic rCD8^+^ T-cells showed greater cytotoxicity than naive and trained splenic CD8^+^ T-cells

**Fig. 5:**
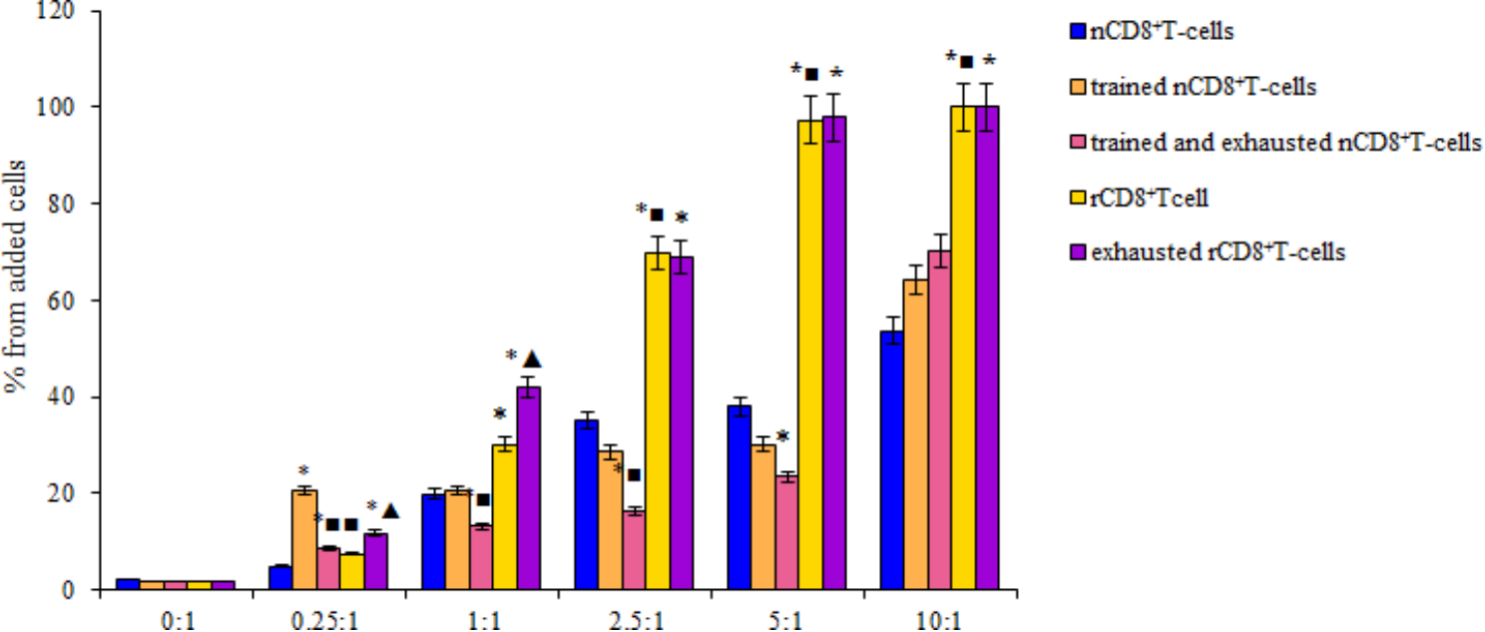
The number of apoptotic tumor LLC cells after co-culturing with splenic naive (nCD8^+^T-cells), trained naive (trained nCD8^+^T-cells), trained and exhausted naive (trained and exhausted nCD8^+^T-cells), reprogrammed (rCD8^+^T-cells), and exhausted reprogrammed (exhausted rCD8^+^T-cells) CD8^+^ T-cells (% from added cells) in ratio between T-lymphocytes and LLC 0.25:1, 1:1, 2.5:1, 5:1, and 10:1. *– for comparison with the naive T-cells, ■– for comparison with trained naive T-cells, ▲– for comparison with reprogrammed T-cells (Mann–Whitney test, *p*-value < 0.05). Data were obtained by analyzing images obtained using Cytation 5 Cell Imaging Multimode Reader, widefield fluorescence microscopy

The results of the in vitro studies presented in Fig. 5 showed that with an increase in the concentration of naive CD8^+^ T-cells, the number of dead LLC in culture increased. Training did not affect the cytotoxicity of CD8^+^ T-cells. Only in the ratio of T-cells: LLC = 0.25: 1 did the cytotoxicity of the trained cells exceed that of the naive ones. Reprogramming increased the cytotoxicity of CD8^+^ T-cells relative to naive cells at concentrations of 1:1; 2.5:1; 5:1; 10:1.

The exhausted procedure did not affect the cytotoxicity of rCD8^+^ T-cells and reduced the cytotoxicity of trained CD8^+^ T-cells at concentrations of 0.25:1; 1:1 and 2.5:1 (Fig. 5).

### Cell therapy with splenic rCD8^+^ T-cells inhibited lung tumor growth and reduced metastatic activity in LLC mice

**Fig. 6:**
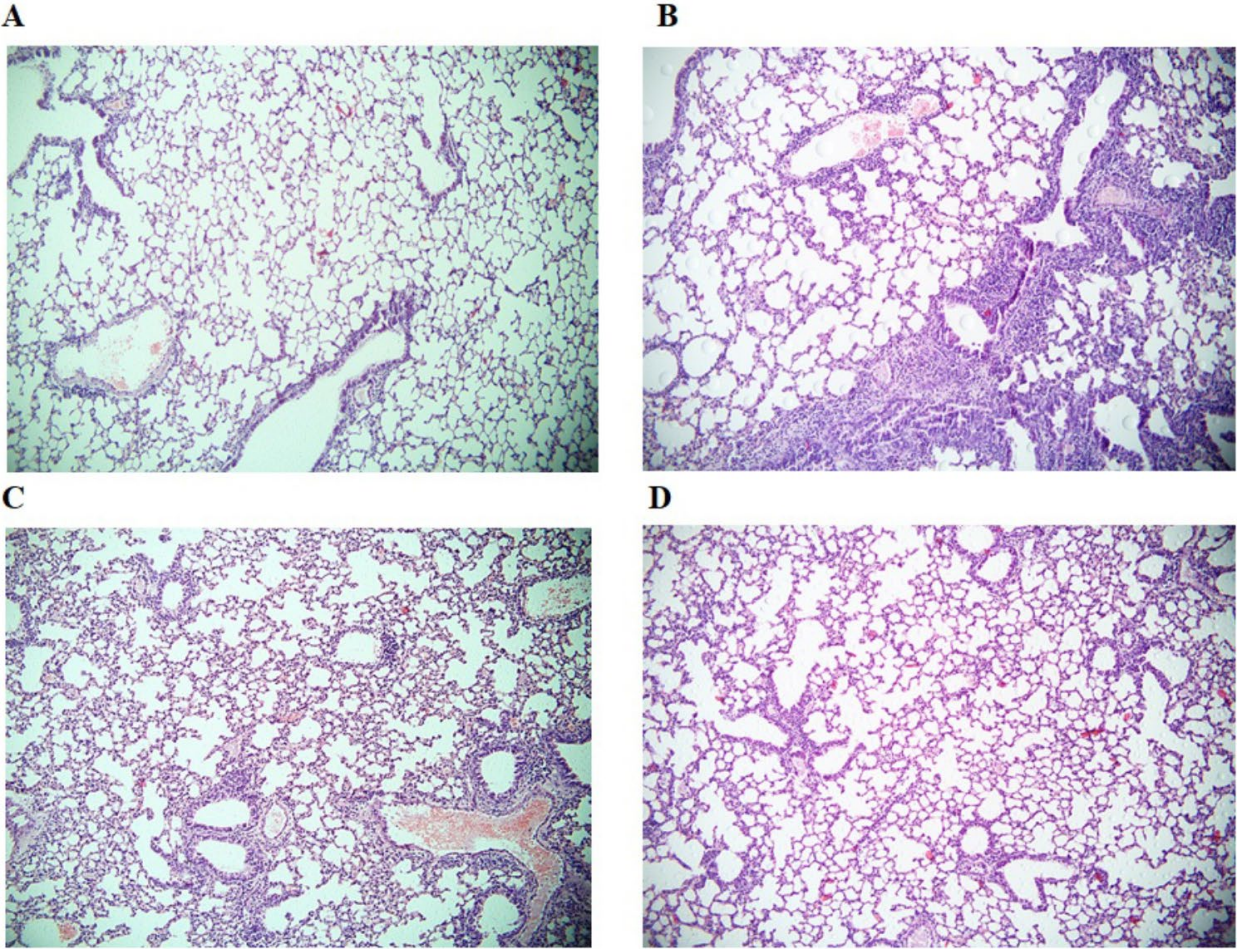
Micrographs of lung sections obtained from C57BL/6 mice of **A** intact control, **B** mice with LLC, **C** mice with LLC treated with naive CD8^+^ T-cells and **D** mice with LLC treated with reprogrammed CD8^+^ T-cells on d10. Tissues were stained with hematoxylin-eosin. ×100

On the 10th day after the injection of LLC cells in the lungs of C57BL/6 mice, reactive edema and inflammatory infiltration of the parenchyma by lymphocytes and macrophages were noted. Multiple metastases were found. Metastases consisted of large cells, round in shape, characterized by severe atypia. This group of cells is characterized by polymorphism of cells and nuclei. Giant multinucleated cells were detected in the population. The chromatin of the tumor cells had a rough clumpy structure, there were many mitotic figures. Metastases were located perivascularly and peribronchially, germinated into the surrounding tissues and leading to their compression and degeneration (Fig. 6). Emboli from tumor cells were observed in large vessels.

Part of the tumor nodes became vascularized, foci of necrosis with perifocal inflammation were noted in the thickness of the node. As can be seen from Fig. 4; Table 1, in the lungs of pathological control mice, tumor and metastatic disease actively progressed.

Administration of naive CD8^+^ T-cells isolated from the spleen did not change the histological pattern in the lungs of mice and did not affect morphometric parameters (tumor weight, tumor volume, mean number of lung metastases) in the LLC spontaneous metastasis model (Fig. 6; Table 3).

Therapy with splenic rCD8^+^ T-cells significantly reduced tumor weight, coefficient of tumor weight, percentage of tumor from body weight and tumor volume. In addition, there was a decrease in the frequency of metastasis, the extent of lung metastasis, and the average number of lung metastases (Table 3). The results of calculating the tumor growth inhibition index (TGII) and the metastasis inhibition index (MII) confirmed the effectiveness of reprogrammed splenic T-lymphocytes to inhibit tumor growth (TGII = 27.8%) and metastasis (MII = 100%).

rCD8^+^T-cells therapy had no effect on the general condition of mice with lung cancer: reduced body weight, forced hunched posture, decreased motor activity, pale gray skin, dull and matted fur.

**Table 3:** Effect of cell therapy of reprogrammed CD8^+^ T-cells of the spleen on tumor and metastases in C57BL/6 mice under conditions of spontaneous metastasis LLC on the 10th day of the experiment (M ± m)

Characteristics	Intact control	LLC	Naive CD8^+^ T-cells	Reprogrammed CD8^+^T-cells
Body weight, g	20.44 ± 0.12	22.73 ± 0.71	22.58 ± 0,59	22.30 ± 0.25
Tumor weight, mg	-	89.25 ± 19.21 ^*^	100.50 ± 18.84 ^*^	52.50 ± 13.52 ^*●+^
Tumor weight ratio, mg/g	-	3.95 ± 0.87 ^*^	4.41 ± 0.75 ^*^	2.36 ± 0.61 ^*^
% of the tumor by body weight	-	0.40 ± 0.09 ^*^	0.44 ± 0.08 ^*^	0.24 ± 0.06 ^*^
Tumor volume, mm^3^	-	130.05 ± 35.32 ^*^	109.38 ± 28.50 ^*^	93.81 ± 28.97
The frequency of metastasis, %	-	100.00 ± 1.00 ^*^	100.00 ± 1.00 ^*^	0 ^**+**^
The degree of lung metastasis	-	1.00 ± 0.01 ^*^	1.00 ± 0.01 ^*^	0 ^**●+**^
Average number of metastases	-	2.75 ± 0.25 ^*^	2.25 ± 0.48 ^*^	0 ^**●+**^
Lung weight, mg	109.6 ± 2.14	80.00 ± 8.61	131.00 ± 9.28 ^*●^	108.8 ± 26.90

*– for comparison with intact control, ●– for comparison with LLC, +– for comparison with naive T-cells (Mann–Whitney test, *p*-value < 0.05)

### In spontaneous metastasizing lung carcinoma, rCD8^+^ T-cells of the spleen have an inhibitory effect on cancer cells and CSC expressing Sox2 and markers Axl, CD117, EGF, PD-L1 and PD-1

Cancer cells and CSCs from various tumors have only been partially characterized phenotypically and functionally. Tissue-specific markers of cancer cells and CSCs have not been unequivocally determined. We have identified frequently used markers for the determination of cancer cells and CSCs: Axl, CD44, CD90, CD117, CD276, EGF, PD-L1, PD-1, Sox2 [22–30].

Axl is a driving force for the extension of tumors in both in vivo and in vitro studies. GAS6/Axl signaling functions as an important pathway governing cancer cell survival, proliferation, migration, and invasion, making Axl a marker of cancer progression and metastasis, and a potential target in cancer therapy [31].

The cell adhesion molecule CD44 is weakly expressed in normal tissues, usually associated with CD133^+^ cells in cancer metastases [32]. One of the cell lines was characterized as CD44^hi^CD90^+^ and this set of markers was proposed for the identification of CSCs [33]. CD44v8-10 is expressed on various human epithelial malignancies, including lung and CSC. CD44v8-10 expression correlates with metastasis [34, 35].

CD117 (Kit, c-Kit) is a type III receptor tyrosine kinase involved in the activation of many intracellular pathways regulating a number of biological processes, such as apoptosis, differentiation, adhesion, and cell proliferation [29]. Overexpression of CD117 is observed in lung cancer. Overexpression of CD117 in lung cancer is also associated with poor prognosis, lower survival, and chemoresistance.

CD276 plays a role in enhancing cancer cell survival by inhibiting natural killer-induced cell lysis [36]. The PD-1/PD-L1 axis is involved in the mechanism of tumor escape from the immune response [37, 38].

The epidermal growth factor receptor (EGFR) is a key factor in epithelial malignancies. EGFR activity enhances tumor growth, invasion, and metastasis [39]. In cancer, EGFR is often continuously upregulated due to sustained production of EGFR ligands in the tumor microenvironment [40] or as a result of a mutation of the EGFR itself, which blocks the receptor in a state of constant activation [41].

Sox2 plays an important role in maintaining adult stem cell stemness. An increase in Sox2 expression is observed in various types of cancer, determining the proliferation, migration, invasion, and metastasis of cancer cells [42]. In general, Sox2 protein expression is associated with aggressive tumors [29, 43, 44]. The worst overall survival in SCLC was found at high levels of Sox2 expression in the CSC [29, 45]. Activation of Sox2 enhances the proliferation of cancer cells and it is important for the function of the lung CSCs [46].

**Fig. 7:**
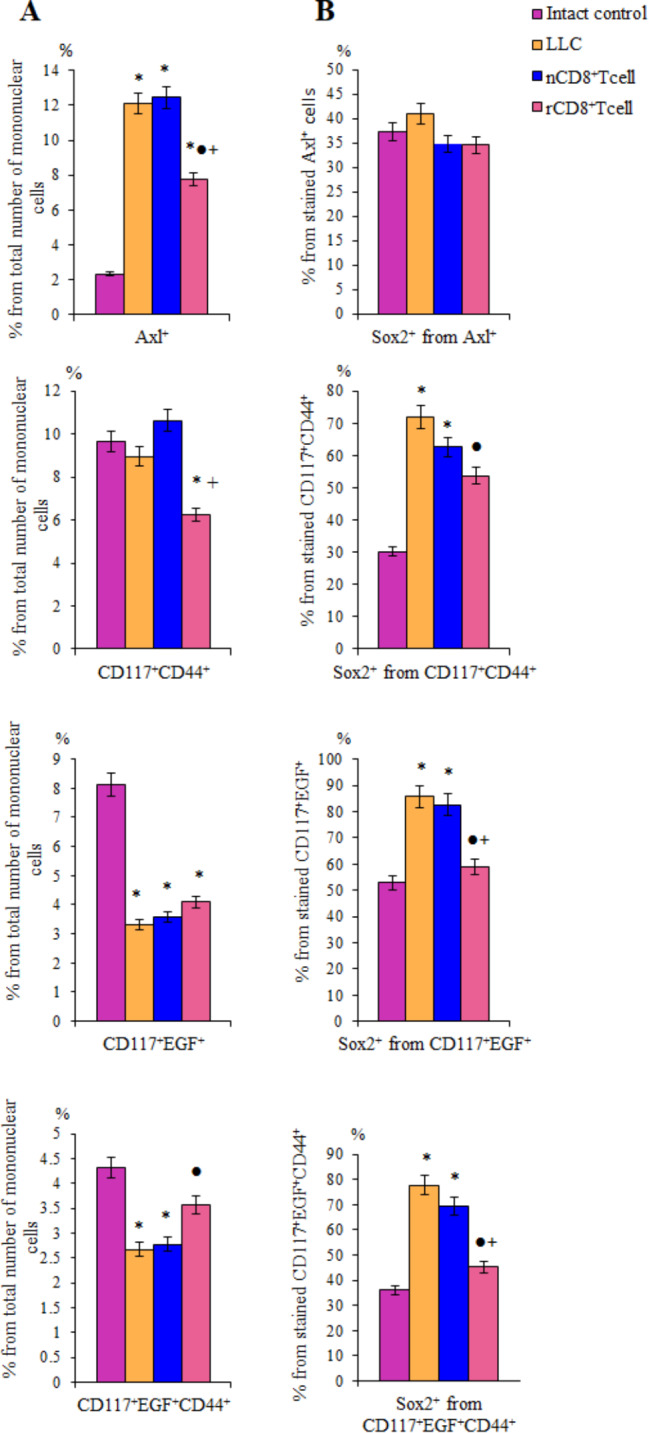
The effect of cell therapy naive and reprogrammed CD8^+^ T-cells on cancer cells and cancer stem cells (CSCs). **A** The number of Axl^+^, CD117^+^CD44^+^, CD117^+^EGF^+^, and CD117^+^EGF^+^CD44^+^ cells in lung mice with LLC, d10 (% of total mononuclear cells number); **B** relative number of Sox2^+^ cells in a population of Axl^+^, CD117^+^CD44^+^, CD117^+^EGF^+^, and CD117^+^EGF^+^CD44^+^ cells (% of stained CSCs). *– for comparison with intact control, ●– for comparison with LLC, +– for comparison with naive T-cells (Mann–Whitney test, *p*-value < 0.05). All data were obtained using flow cytometry

**Fig. 8:**
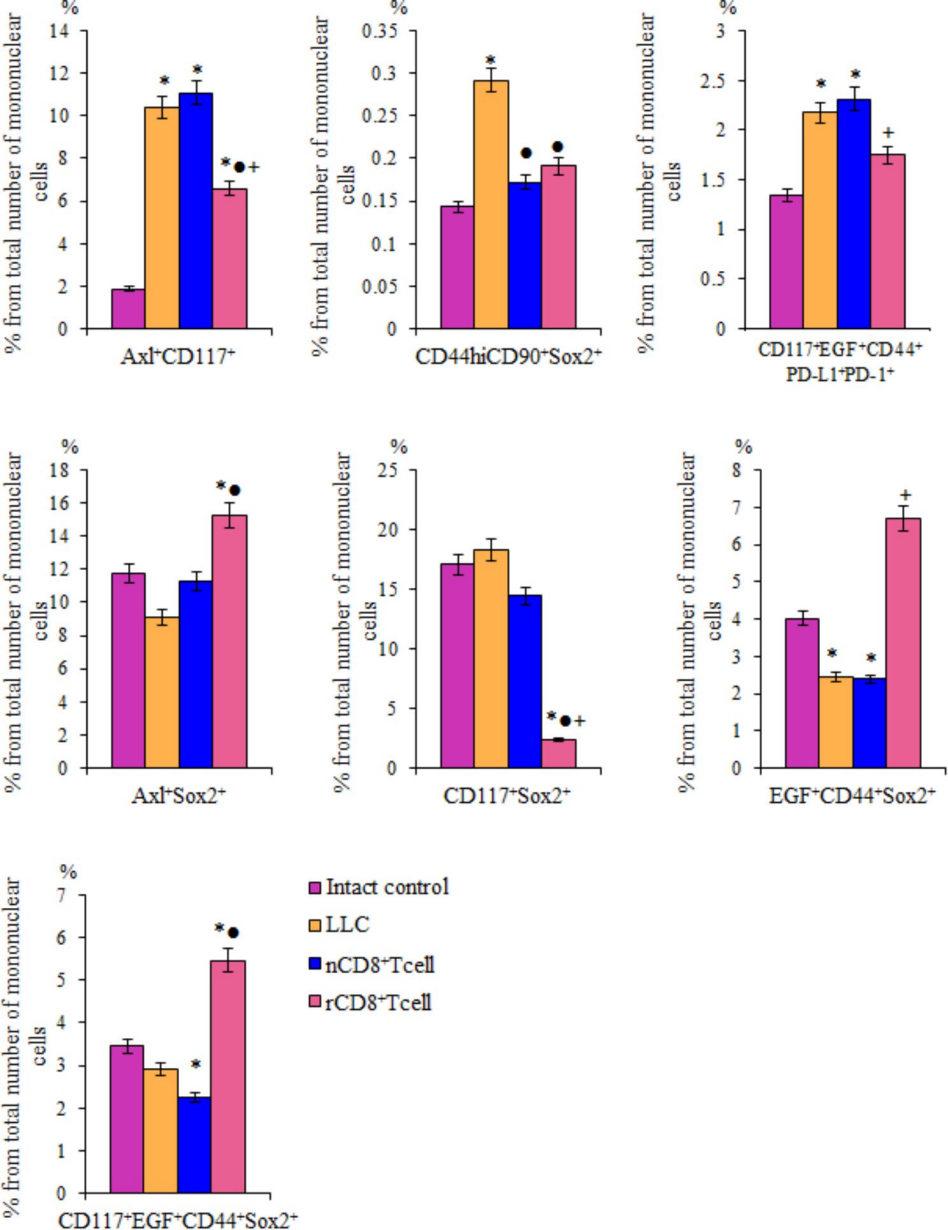
The effect of cell therapy naive (nCD8^+^T-cells) and reprogrammed (rCD8^+^T-cells) CD8^+^ T-cells on number (% of total mononuclear cells number) of Axl^+^CD117^+^, CD44^hi^CD90^+^Sox2^+^, CD117^+^EGF^+^CD44^+^PD-L1^+^PD-1^+^, Axl^+^Sox2^+^, CD117^+^Sox2^+^, EGF^+^CD44^+^Sox2^+^, and CD117^+^EGF^+^CD44^+^Sox2^+^ cells in lung mice with LLC, d10. *– for comparison with intact control, ●– for comparison with LLC, +– for comparison with naive T-cells (Mann–Whitney test, *p*-value < 0.05). All data were obtained using flow cytometry

Along with histopathological changes in the lungs in mice with LLC (Fig. 6), we found an increase in the content in the lungs of a number of CSCs with the phenotype Axl^+^, Axl^+^CD117^+^, CD117^+^EGF^+^CD44^+^PD-L1^+^PD-1^+^ (Figs. 7a and 8). In addition, an increase in the number of populations expressing Sox2: CD117^+^Sox2^+^, CD44^hi^CD90^+^Sox2^+^ was observed. Despite the fact that the absolute number of CD117^+^CD44^+^, CD117^+^EGF^+^ and CD117^+^EGF^+^CD44^+^ cells did not increase in tumor formation, the relative content of Sox2^+^ cells in these populations was significantly higher compared to similar indicators in intact animals (Fig. 7b). Expression of the Sox2 protein is associated with more aggressive tumors. Thus, with these cells, we associate the development of a tumor and metastatic disease in the lungs of C57Bl/6 mice after a single subcutaneous injection of LLC into the right axillary region. Cell therapy with rCD8^+^ T-cells reduced the amount of Axl^+^, Axl^+^CD117^+^, CD117^+^ EGF^+^CD44^+^PD-L1^+^PD-1^+^ and CD117^+^CD44^+^ cells in the lungs of mice with spontaneously metastatic tumor (Figs. 7a and 8). At the same time, the number of cells with the immunophenotype CD117^+^Sox2^+^, CD44^hi^CD90^+^Sox2^+^ also decreased. In parallel with this, in the CD117^+^CD44^+^, CD117^+^EGF^+^ and CD117^+^EGF^+^ CD44^+^ populations, the content of Sox2^+^ cells decreased (Figs. 7a and 8).

Therapy with naive CD8^+^ T-cells had a controversial effect on the lung of mice with spontaneous metastasizing tumors. Specifically, the number of CD44^hi^CD90^+^Sox2^+^, CD117^+^Sox2^+^ cells reduced and the number of CD117^+^CD44^+^, Axl^+^CD117^+^ cells slightly increased (Figs. 7a and 8). The content of cells with a phenotype Axl^+^, CD117^+^EGF^+^, EGF^+^CD44^+^Sox2^+^, CD117^+^EGF^+^CD44^+^ Sox2^+^ and CD117^+^EGF^+^CD44^+^PD-L1^+^PD-1^+^ remained unchanged at a high level.

### Administration of splenic rCD8^+^ T-cells increased the content of various populations of CD8^+^ T-cells in the lungs in spontaneous metastasizing lung carcinoma

**Fig. 9:**
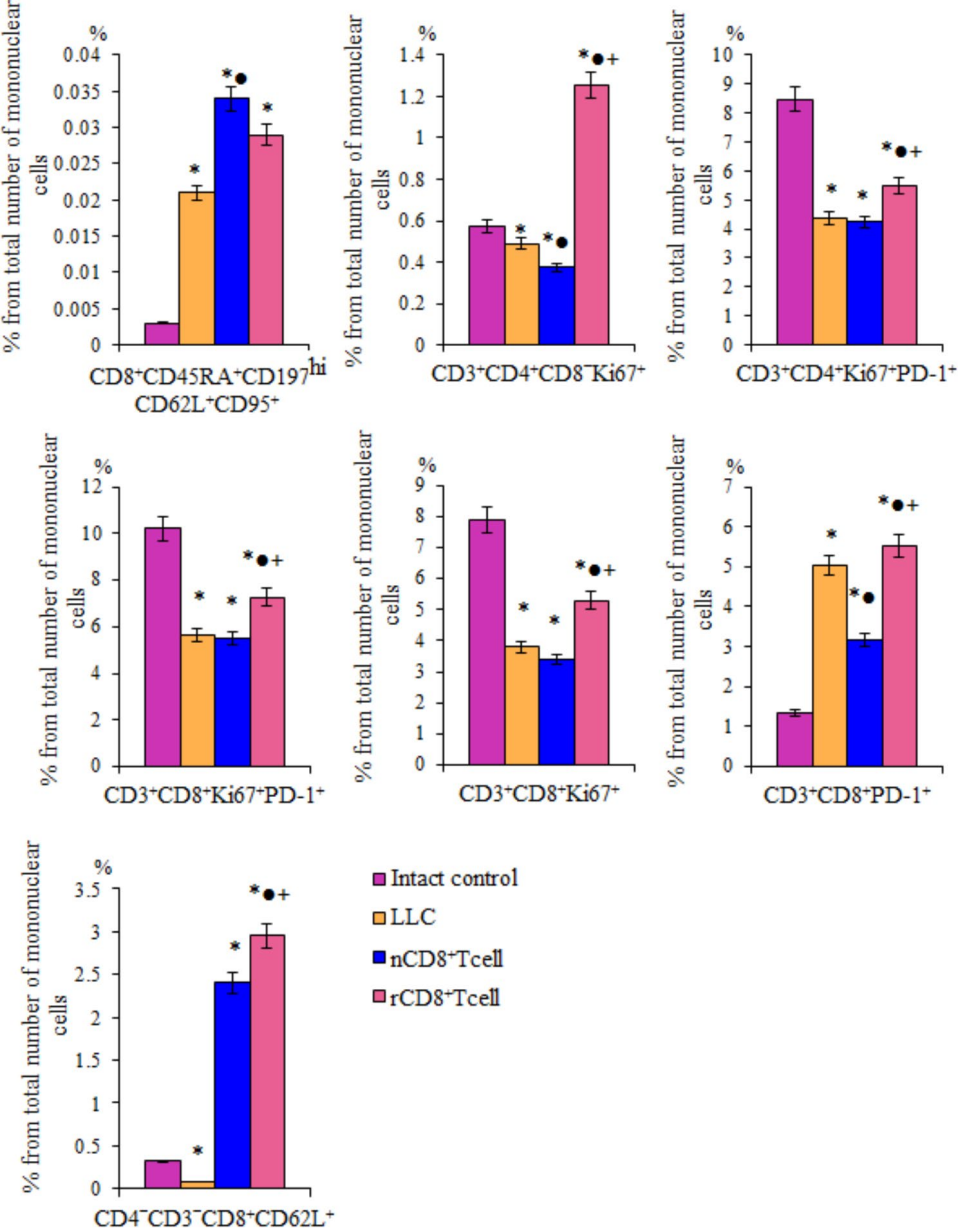
The effect of cell therapy naive (nCD8^+^T-cells) and reprogrammed (rCD8^+^T-cells) CD8^+^ T-cells on CD8^+^CD45RA^+^CD197^hi^CD62L^+^CD95^+^, CD3^+^CD4^+^CD8^−^Ki67^+^, CD3^+^CD4^+^Ki67^+^PD-1^+^, CD3^+^CD8^+^Ki67^+^PD-1^+^, CD3^+^CD8^+^Ki67^+^, CD3^+^CD8^+^PD-1^+^, CD4^−^CD3^−^CD8^+^CD62L^+^ T-cells in lung mice with LLC, d10. *– for comparison with intact control, ●– for comparison with LLC, +– for comparison with naive T-cells (Mann–Whitney test, *p*-value < 0.05). All data were obtained using flow cytometry. Characteristics of the T-cell populations are presented in Supplementary Table 2

CD8^+^ T-cells are the main cells of antitumor immunity. In the lungs of mice with metastasizing LLC, the content of CD8^+^CD45RA^+^CD197^hi^CD62L^+^CD95^+^ Т-cells with low regenerative potential, CD4^‒^CD3^‒^CD8^+^CD62L^+^ Т-cells and activated CD3^+^CD8^+^PD-1^+^ Т-cells increased (Fig. 9). At the same time, the number of T-cells with the phenotype CD3^+^CD8^+^Ki67^+^ and CD3^+^CD8^+^Ki67^+^PD-1^+^ decreased. CD4^+^ T-cells are involved in the antitumor immune response [47]. Number of populations of CD4^+^ Т-cells (CD3^+^CD4^+^CD8^‒^Ki67^+^ and CD3^+^CD4^+^Ki67^+^PD-1^+^) studied by us decreased in the lungs of animal with LLC (Fig. 9).

Administration of rCD8^+^ T-cells to LLC mice increased T-cells, which were reduced in response to tumor formation. Thus, in the lungs of mice, the content of cells with the phenotype CD3^+^CD8^+^Ki67^+^, CD3^+^CD8^+^Ki67^+^PD-1^+^, CD3^+^CD4^+^CD8^‒^Ki67^+^, CD3^+^CD4^+^Ki67^+^PD-1^+^ increased (Fig. 9). At the same time, cell therapy did not affect the high level of CD3^+^CD8^+^PD-1^+^ и CD8^+^CD45RA^+^CD197^hi^CD62L^+^CD95^+^ Т-cells. The number of CD4^‒^CD3^‒^CD8^+^CD62L^+^ T-cells in response to therapy increased even more.

After the injection of nCD8^+^ T-cells, the content of CD8^+^CD45RA^+^CD197^hi^CD62L^+^CD95^+^ Т-cells increased (Fig. 9). Cell therapy reduced the number of Т-cell with phenotype CD3^+^CD4^+^CD8^−^Ki67^+^ and CD3^+^CD8^+^PD-1^+^ and did not affect T-cells with phenotype CD3^+^CD4^+^Ki67^+^PD-1^+^, CD3^+^CD8^+^Ki67^+^, CD3^+^CD8^+^Ki67^+^PD-1^+^ and CD4^‒^CD3^‒^CD8^+^CD62L^+^.

## Discussion

In the present study, we continued to study the effects of reprogrammed CD8^+^ T-cells in various models of lung cancer in vitro and in vivo. We evaluated the antitumor and antimetastatic effects of CD8^+^ T-cells isolated from the spleen of C57Bl/6 mice. To increase the antitumor activity of CD8^+^ T-cells, we performed reprogramming using a MEK inhibitor in combination with a PD-1 blocker. Previously, Verma V. et al. showed that inhibition of the MAPK signaling pathway by MEK1/2i causes the formation of T memory stem cells (TSCM) from effector CD8^+^ T-cells. TSCM are resistant to the loss of surface markers and retain their functions when depleted [48]. In more detail, the mitogen-activated protein kinase (MAPK) signaling pathway transmits external mitogenic signals to immune cells. This results in modulation of cellular differentiation and the metabolic machinery that prepares cells for effector functions. Constant mitogenic stimulation of effector cells, which occurs under the influence of the tumor microenvironment, leads to exhaustion of T-cells, a decrease in their effector functions and impaired immune memory. MEK inhibition suppresses cyclin D1, which leads to cell cycle inhibition and improves metabolic activity by modulating the ERK1/2-cyclin D1-PGC1α SIRT3-FAO pathway, which promotes the formation of memory stem cell-like cells (T_SCM_) [48]. At the same time, detailed molecular mechanisms of - cell differentiation and functions upon MEK inhibition require further study.

PD-1 is a negative regulator of T-cell activity. The PD-1/PD-L1 signaling pathway is an important component of tumor immunosuppression and promotes cancer escape from immune surveillance [49]. Blockade of PD-1 on CD8^+^ T-cells contributes to the restoration of their cytotoxic function [50]. For this reason, we additionally used the PD-1 blocker nivolumab [51]. Selective cytotoxicity in relation to cancer cells was achieved by “training” the population of T-lymphocytes with a CSC lysate. Expression of the chemokine receptor CCR7 after reprogramming was higher than in naive CD8^+^ T-cells. The expression of CCR7 by reprogrammed CD8^+^ T-cells remained at a high level even after exhaustion (Fig. 3). As previously shown by Verma V. et al., decreased CCP7 expression is characteristic of exhausted T-cells [48]. Thus, MEKi and nivolumab-induced changes in rCD8^+^ T-cells of the spleen were persistent even in presence of the immunosuppressive action of the tumor.

CD8^+^ T-cells were chosen due to the active migration of naive and reprogrammed populations into the lungs of C57BL/6 male mice after injection into the tail vein. We observed the maximum values at the end of the first hour of observation. This was not observed after injection of naive and reprogrammed CD4^+^ T-cells (data not shown).

At its initial stage, lung cancer often lacks pronounced symptoms. In the advanced stage, the cancer metastasizes extensively. In the presence of distant metastases, treatment comprises of palliative care, chemotherapy, immunotherapy and radiation therapy [52]. The effectiveness of treatment is still insufficient. We evaluated the antitumor and antimetastatic effects of reprogrammed splenic CD8^+^ T-cells in the LLC metastatic model. We evaluated the efficacy of reprogrammed CD8^+^ T-cells in vitro. The survival and cytotoxicity of rCD8^+^ T-cells in LLC culture was significantly higher in comparison with naive CD8^+^ T-cells (Figs. 4 and 5).

A course of cell therapy with rCD8^+^ T-cells isolated from the spleen caused the accumulation of various subpopulations of CD8^+^ T-cells in the lungs of mice with LLC. CD4^+^ T-cells are known to be involved in antitumor immunity. They cooperate with CD8^+^ T-cells as well as have their own cytotoxic activity [53]. We found a significant increase in various populations of CD4^+^ T-cells in the lungs of mice with spontaneously metastasizing LLC after cell therapy. At the same time, the content of CD8^+^ T-cells increased. In response to this, the content of CSC in the lungs decreased. Although identification of a specific tumor-specific epitope is difficult due to the phenotypic diversity of cancer cells, all these data suggest that CSCs were targeted by T-cells. This explains the partial reduction of the tumor in the lungs and a decrease in the frequency of metastasis.

Monitoring the condition of animals did not reveal the effect of cell therapy on body weight, mice posture, motor activity, skin and fur or other side effects. However, we understand the limitations of our study. Additional studies are required to evaluate the side effects of cell therapy.

## Conclusions

T-cell reprogramming by inhibiting the MAPK/ERK signaling pathway through MEKi and the use of an immune checkpoint blocker increases the antitumor activity of splenic CD8^+^ T-cells in a spontaneously metastatic LLC model. At the same time, cell therapy with rCD8^+^ T-cells of the spleen has an inhibitory effect on lung cancer cells and CSCs expressing Sox2 and markers Axl, CD117, EGF, PD-L1 and PD-1. Our method for reprogramming splenic CD8^+^ T-cells may be useful in developing an approach to the treatment of metastatic disease in patients with lung cancer.

### Electronic supplementary material

Below is the link to the electronic supplementary material.

Supplementary Material 1

## Data Availability

The datasets analysed during the current study are available from the corresponding author on reasonable request.

## Abbreviations

CCR7: C-C chemokine receptor type 7
CSCs: cancer stem cells
nCD8^+^T-cells: naive CD8^+^ T-cells
rCD8^+^T-cells: reprogrammed CD8^+^ T-cells
