# Supplementary material for: Effects of reprogrammed splenic CD8+ T-cells in vitro and in mice with spontaneous metastatic Lewis lung carcinoma
Source: BMC Cancer. 2024 Apr 25;24:522. doi: 10.1186/s12885-024-12203-y (PMC11046928; doi:10.1186/s12885-024-12203-y)
Supplement: Supplementary file 1 — Supplementary Material 1 [file 12885_2024_12203_MOESM1_ESM.pdf]

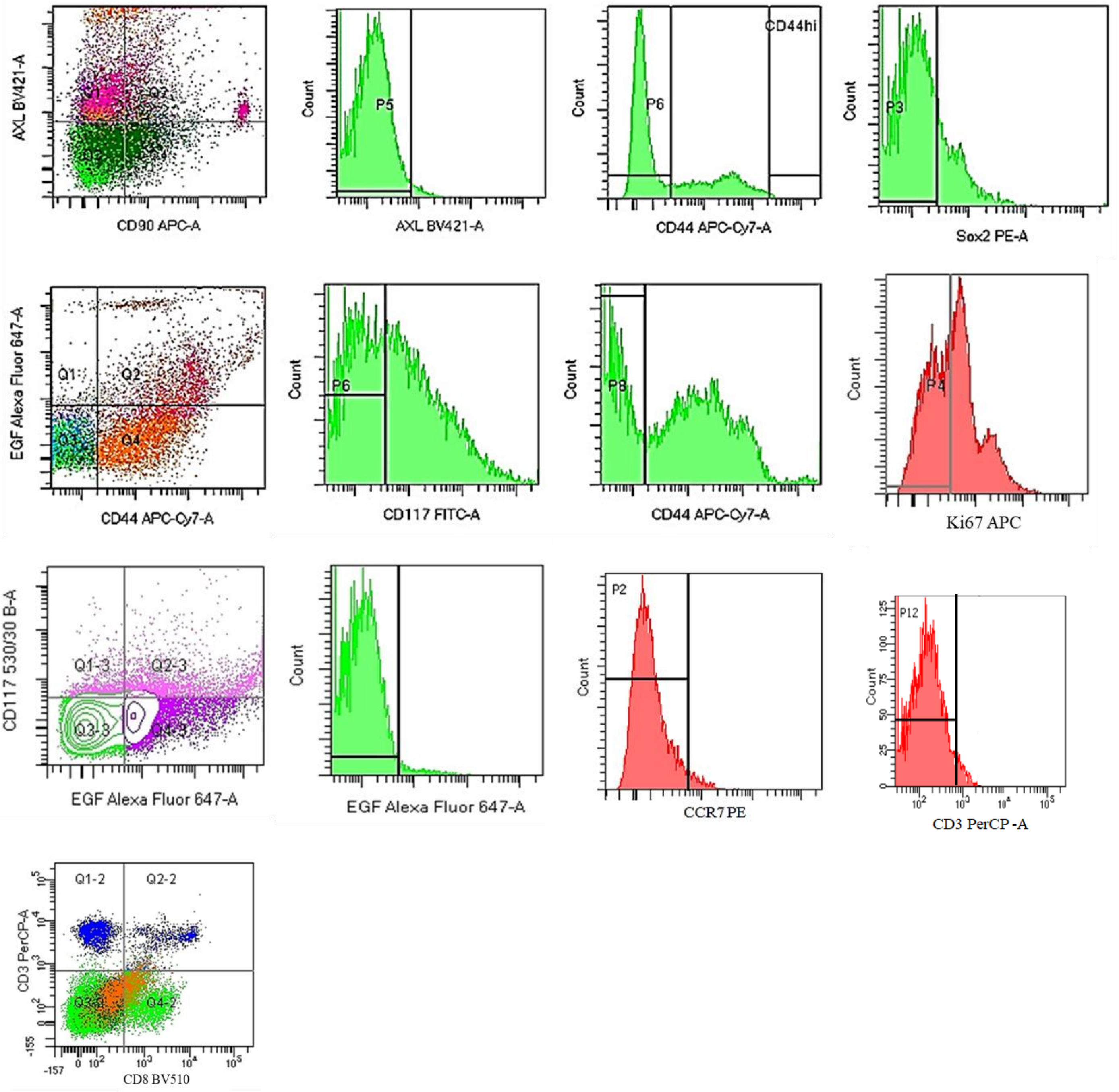

Supplementary Figure 1. Flow cytometry images representing phenotype establishment and qualitative analysis of CD90 (APC), Axl (BV421), CD44 (APC-Cy™7), Sox2 (PE), EGF/CD44, CD117 (FITC), Ki67 (APC), EGF (Alexa Fluor® 647), CCR7 (PE), CD3 (PerCP), CD8 (BV510).
